# Strategies for Improving Antimicrobial Properties of Stainless Steel

**DOI:** 10.3390/ma13132944

**Published:** 2020-06-30

**Authors:** Matic Resnik, Metka Benčina, Eva Levičnik, Niharika Rawat, Aleš Iglič, Ita Junkar

**Affiliations:** 1 Department of Surface Engineering and Optoelectronics, Jožef Stefan Institute, Jamova 39, SI-1000 Ljubljana, Slovenia; metka.bencina@ijs.si (M.B.); eva.levicnik@ijs.si (E.L.); ita.junkar@ijs.si (I.J.); 2 Laboratory of Physics, Faculty of Electrical Engineering, University of Ljubljana, Tržaška 25, SI-1000 Ljubljana, Slovenia; niharika.rawat@fe.uni-lj.si (N.R.); ales.iglic@fe.uni-lj.si (A.I.); 3 Faculty of Medicine, University of Ljubljana, Zaloška 9, SI-1000 Ljubljana, Slovenia

**Keywords:** antibacterial, stainless steel, surface modification, biocompatibility, plasma

## Abstract

In this review, strategies for improving the antimicrobial properties of stainless steel (SS) are presented. The main focus given is to present current strategies for surface modification of SS, which alter surface characteristics in terms of surface chemistry, topography and wettability/surface charge, without influencing the bulk attributes of the material. As SS exhibits excellent mechanical properties and satisfactory biocompatibility, it is one of the most frequently used materials in medical applications. It is widely used as a material for fabricating orthopedic prosthesis, cardiovascular stents/valves and recently also for three dimensional (3D) printing of custom made implants. Despite its good mechanical properties, SS lacks desired biofunctionality, which makes it prone to bacterial adhesion and biofilm formation. Due to increased resistance of bacteria to antibiotics, it is imperative to achieve antibacterial properties of implants. Thus, many different approaches were proposed and are discussed herein. Emphasis is given on novel approaches based on treatment with highly reactive plasma, which may alter SS topography, chemistry and wettability under appropriate treatment conditions. This review aims to present and critically discuss different approaches and propose novel possibilities for surface modification of SS by using highly reactive gaseous plasma in order to obtain a desired biological response.

## 1. Introduction

Metallic biomaterials, especially stainless steel and titanium alloys, are one of the most widely used materials for biomedical application, as they possess excellent mechanical properties and biocompatibility. Stainless steel (SS) is the iron-based alloy containing at least 10.5% of chromium (Cr), which allows for corrosion prevention and improves mechanical properties [1]. First SS implants were employed for bone implants in 1920s [2] and were also rapidly used in other applications. Currently SS materials are used for permanent (bone implants, stents, dental implants etc.) and temporary implants (plates, nails, screws etc.) as well as for surgical tools (scalpels, guiding templates etc.). This is mainly due to its comparatively low cost, ease of manufacturing, good fatigue properties and reasonable corrosion resistance and biocompatibility. It is estimated that nearly 60% of surgical implants used in the United States are made of SS [3], while approximately 85% of surgical instruments are made of SS [4]. However, exposure of SS to harsh biological environment in the human body may lead to undesired surface interactions like biofilm formation and corrosion. Moreover, SS surfaces do not possess desired bio-functional properties like hemocompatibility (cardiovascular implants) or osteoblast proliferation (bone implants, dental implants), and do not prevent bacterial adhesion and biofilm formation. This dictates the use of various surface finishing procedures (coatings, biofunctionalization methods etc.) which influence surface properties and should preserve the bulk attributes of material at the same time (mechanical properties). Although SS is a widely used biomaterial, there are still limited amounts of available reviews that critically present various types of surface finishing strategies for the prevention of bacterial adhesion and biofilm formation on SS. The main aim of the present review is aimed at this issue in particular, as it critically assesses various types of treatment strategies and presents innovative possibilities which may provide novel approaches that enable fabrication of antibacterial surfaces based on SS material. In the first section, the use of SS materials in biomedical applications is presented and its benefits and constraints are discussed. Further on, discussion on bacterial infections and the biological response of SS surfaces used for specific applications is presented (surgical tools, cardiovascular implants, bone implants), and known strategies used for bacterial prevention are discussed. Due to a large increase in antibiotic resistant bacterial strains, bacterial infections present a serious health care concern, meaning more appropriate surface modification strategies and knowledge on surface induced bacterial interaction should be obtained and employed. Thus, the second part of the review deals with the most important surface features influencing the biological responses of SS, like surface chemistry, morphology and wettability/surface charge. Findings of different studies used for surface modification are presented and discussed. At the end of this review, strategies for surface modification of SS involving different types of plasma technologies are presented and examples of surface modification of SS 316L obtained using highly reactive radiofrequency plasma are given and discussed.

## 2. The Use of SS in the Biomedical Field

Metal and its alloys are the most popular materials in the biomedical field, and are mainly employed for orthopedic implants due to their appropriate mechanical properties, biocompatibility and good chemical and mechanical stability. The main research in biomaterials engineering is focused on enhancing or replacing the normal functions of biological systems with ideal biomaterials, which provide optimal surface characteristics as well as mechanical properties that enables their use for specific application. In orthopedics, for example, the main issue is to improve osseointegration (formation of bone growing cells) and prevent bacterial adhesion. It should be emphasized that the antibacterial properties are one of the key issues to be considered in all implantable materials (regardless to of final application), thus various strategies for surface modification of biomaterials are used to decrease/prevent bacterial adhesion and biofilm formation [5]. It is quite difficult to design surfaces of biomaterials with desired bulk mechanical properties and appropriate biological responses at the same time. An optimal biomaterial in orthopedics should have appropriate mechanical properties, stimulate growth of bone forming cells (osteoblasts) and prevent bacterial adhesion. It is well known that the highest risk of bacterial infections on implantable material occurs during the surgery (increasing with the time of surgery) and during the first 24 h after surgery. In order to prevent bacterial adhesion, it is imperative to understand the mechanisms and interactions between the host, microorganisms and biomaterials [6,7].

The metallic biomaterials in orthopedic applications require specific mechanical properties for joint replacement, realigning bone fragments and promoting fracture integrity and should at the same time offer long implant lifespan. The hazard of implanting metallic biomaterials into a corrosive biological environment may lead to physiological changes involving implants and corrosion [6] This process affects biocompatibility as well as the mechanical properties of a biomaterial [5]. Corrosion can induce significant damage to the implant and shorten the implant’s lifespan. Corrosion of the biomaterial surface is promoted by adhesion and growth of bacteria, causing chronical microbial contamination and therefore huge post-surgical complications which are dangerous to patients and increase healthcare costs [1]. Due to biomaterial tissue interaction, denaturation of proteins may occur, which further leads to cellular damage and implant failure.

The major concern after implantation is infection, which is caused by the undesired interaction between biomaterial and host tissue. The ultimate goal for biomedical implants is a complete implant integration with no post-surgical complications and adverse foreign body responses, such as chronic inflammatory response or formation of undesired tissue [8]. The bacterial adhesion and biofilm formation can be very difficult to remove, especially due to antibiotic resistant bacterial strains. The prolonged antimicrobial therapy can result in revision surgery and implant removal, which can be life threatening for patients. Thus it is highly important to develop biomaterial surfaces which would prevent bacterial invasion [9]. There are two major factors causing implant failure: (i) infection and (ii) aseptic loosening [10].

Factors contributing to the physicochemical and molecular interactions are defined by bacterial characteristics, the environment (pH, temperature, duration of exposure) and surface properties (wettability, surface topography, roughness, surface chemistry and surface energy). Another important factor is the ability of a biomaterial to produce ions, which influence the growth of bacteria and biofilm. The biofilm of bacteria is a complex community of proliferated bacteria from exopolysaccharide matrix on the surface that are protected from any environmental and physical removal, thereby offering resistance to antibiotics and other defense mechanisms [11]. Most antibacterial protocols include two different types of bacteria: Gram positive and Gram negative bacteria, with the difference between the two in the thickness of the peptidoglycan layer and the presence or absence of the outer lipid membrane. Because of specific wall structure, Gram positive bacteria is less resistant than Gram negative bacteria. Usually *Staphylococcus aureus* (Gram-positive) and *Escherichia coli* (Gram-negative) bacteria are used to test the antibacterial properties of biomaterials. *S. aureus* can be methicillin resistant (MRSA), which is a major cause of infections, leading to implant failure [12]. It is very important to understand the factors controlling microbial adhesion and retention on metallic surfaces.

**Figure 1:**
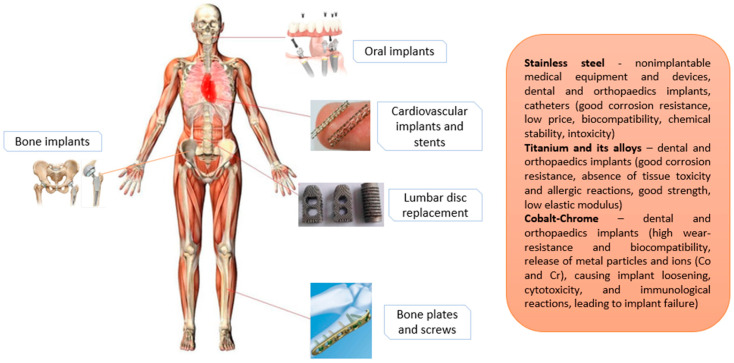
Examples of the use of metallic biomaterials in the human body.

The most common metallic biomaterials used in the implantable biomedical field are stainless steel, titanium and its alloys, cobalt-chromium, tantalum, silver and zirconia. In Figure 1, examples of metallic implants used in the human body are presented.

Depending on the application, different types of materials are employed. For example, titanium and its alloys are one of the most desirable biomaterials used for implants, especially in orthopedics [7,13] and dental implants [6]. Its long term stability and ability to effectively integrate with bone makes titanium and its alloys ideal biomaterials [14]. Furthermore, Ti-based biomaterials have some attractive characteristics suitable for implants, such as low density, heat resistance, corrosion and ductility which reduces the problems associated with stress shielding [15]. Ti-based alloys are mostly used for bone fragments, due to their relatively low elastic modulus.

Co-Cr alloys are commonly employed in vascular stents, orthopedics and dental applications, but are more prone than Ti and its alloys to elicit allergic or immunogenic reactions. This is mainly triggered by the tribological effect where metal ions and particles from Co-Cr alloys are released in the host body, causing implant loosening, immunological failure and cytotoxicity [16]. Moreover, Co-Cr-Mo alloys exhibit excellent anti-corrosion characteristics in artificial joint prostheses [6].

SS is the iron-based biomaterial alloy, low carbon steel contains various elements such as a minimum chromium (Cr) content of 10.5%, which improves corrosion resistance and mechanical properties [1], and is used for medical equipment, devices and implants. Cr provides for corrosion-resistance properties, thus by using increased Cr content, corrosion resistance and other useful properties can be enhanced. In biomedical engineering, SS is mostly used as implant material due to its biocompatibility, good mechanical properties, chemical stability, non toxicity and accessibility [5]. Thus SS is one of the common, widely used materials for various demanding applications such as bone implants, oral implants, cardiovascular implants and stents, for lumbar disc replacement, bone plates and screws (as seen on Figure 1), hospital furniture, surgical equipment and devices, intramedullary nails and external fixation devices [17,18]. Favorable abilities of SS are resistance to corrosion and high temperatures—meaning SS can be sterilized by thermal processes without corroding or losing mechanical and physical properties.

**Figure 2:**
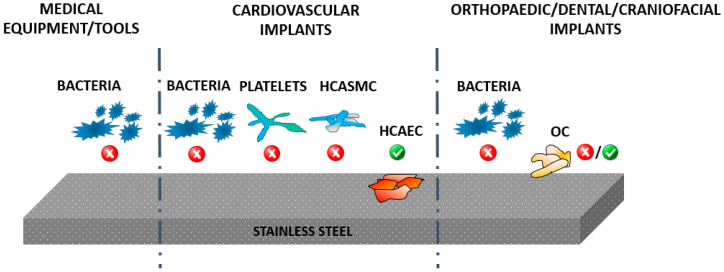
Schematic representation of the applications of stainless steel in medicine and desired response of bacteria and human cells (platelets, HCAEC—human coronary artery endothelial cells, HCASMC—human coronary artery smooth muscle cells, OC—osteoblast cells).

Although SS possess satisfactory biocompatibility and mechanical properties, there are some arising imperfections at the implant surface mainly due to the inhomogeneity of its microstructure. The surface properties of the implants like surface roughness [7,19], chemical composition [20], wettability [21], electrical properties [7,19] and protein adhesion [22] significantly influence bacterial response as well as cell integration. In Figure 2, a schematic representation of SS material used for different medical applications is presented together with desired biological responses. Thus for the case of medical equipment and tools, the main issue is to prevent bacterial adhesion, while the surface should prevent bacterial adhesion in the case of cardiovascular implants, but should at the same time also promote endothelial cell growth, prevent platelet adhesion and reduce the proliferation of smooth muscle cells. While bacterial adhesion is desired in the case of SS materials used in orthopedics prevention, in this case a good proliferation of osteoblast cells (bone forming cells) is also needed in order to assure desired biocompatibility. Thus, surfaces should be appropriately conditioned for their use in specific applications. In all cases, bacterial adhesion should be prevented without influencing the desired biological responses with other cells. The schematic representation of desired events taking place on the SS biomaterial surfaces used for medical tools, cardiovascular implants and orthopedic and dental implants are presented in Figure 2.

The surface of SS is passivated, so there is virtually no exchange of ions with the environment, meaning the surface itself is inert and does possess resistance to forming micro-organisms [23]. Active surfaces, in contrast, release large amounts of metal ions into the environment, which may reduce bacterial adhesion. In case of SS, a very thin layer of oxide film is formed on the surface, which is important to prevent the surface from corroding. However, SS can be produced in several different grades, which affect different psycho-chemical properties of the surface.

SS is classified into four structure grades [24,25]:

1. Martensitic (the hardest crystal structure, magnetic, contains Cr (12–14%), Mo (0.2–1%), 0.1–1% C and no Ni. Extremely strong and tough, it can be hardened by heat treatment and is highly machinable. It is not as corrosion resistant as other grades) [26].
2. Ferritic (overall it contains Cr (10.5–27%) and a low amount of Ni, if any).
3. Austenitic (max. of 0.15% C, min. of 16% Cr and sufficient Ni and/or Mn to retain an austenitic structure at all temperatures. Better resistance to stress-corrosion cracking with a higher Ni content and nitrogen additions).
4. Duplex (has two microstructure phases—ferrite and austenite, with high Cr (19–28%) and Mo (up to 5%) and lower Ni contents than austenitic SS. Alloys have higher strength and greater resistance to localized corrosion, particularly pitting, crevice corrosion and stress corrosion cracking due to chlorides).

**Figure 3:**
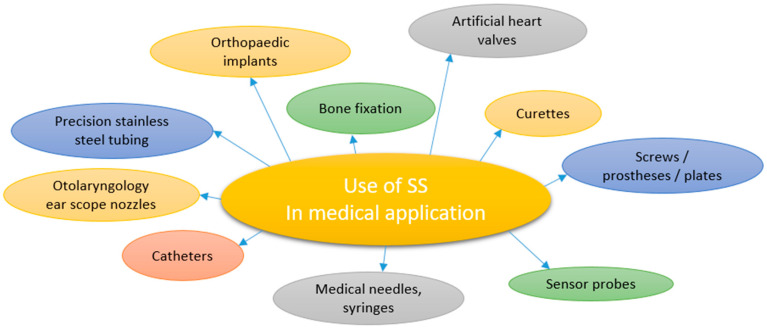
Possible medical device applications of stainless steel.

The first three grades are important in medical applications and implants [27]. For surgical stainless steel, specific grades of SS 316 and 316L are predominantly used. The SS 316 grade is a group of austenitic SS with improved corrosion resistance when in direct contact with biological fluid, superior strength, ductility and toughness to low-alloy steels, due to the addition of Cr (16–18%), Mo (2–3%) and higher Ni content (10–14%). The SSs with the letter ‘L’ such as type 316L have low C content (<0.03%), and are referred as marine grade SSs. SS’s primary alloying constituents after Fe are Cr (16–18%), Ni (10–12%) and Mo (2–3%), with a small (<1%) Si content. Cr, Ni and Mo content in SS offers some unique qualities. Cr is desired for cleaning and sterilization, as it improves the material’s scratch resistance and corrosion resistance. Ni provides an extremely smooth and polishable surface. Mo provides greater corrosion resistance and improved hardness, which makes it great for sharp cutting edges, such as surgical scalpels. As a result of superior mechanical properties, 316 has been the preferred stainless steel alloy, and is recommended for medical devices [28] such as coronary stents, hip-implant stems and spinal-disc replacements. SS is also used for a variety of surgical tools such as scalpels and forceps, as well as for operating tables. Examples of the use of SS 316 and 316L are presented in Figure 3.

Metallic biomaterials like Ti and/or Ti-based alloys have been intensively studied for surface modification; however, there is only a limited amount of studies dealing with surface modification of SS. Thus it is essential to acquire more studies dealing with surface modification of SS for altering antimicrobial activity, interaction with various types of cells and whole blood [8].

## 3. Bacterial Infections

Hospital surfaces are a chronic source of nosocomial pathogens and infections. An important task is to reduce infections by designing antimicrobial surfaces. SS is the most common metallic biomaterial used in hospitals due to ease of cleaning it, its durability and its appearance. Since SS is inert, it does not have sufficient antimicrobial properties for more demanding applications and surface modification must be performed. There are few microorganisms that can persist on surfaces for several months like *Clostridium difficile* spores and methicillin-resistant *Staphylococcus aureus* (MRSA) [28]. In order to reduce the contamination of surfaces, more extensive guidelines and standardized methods must be developed to achieve reliable, reproducible results for antibacterial activity.

One of the problems of microbial contamination of SS surfaces is the promotion of corrosion from bacterial adhesion and growth. Surface topography, chemistry and roughness can inhibit the adhesion of bacteria. Microbial activity depends on physico-chemical properties on a biomaterial surface, the microbial surface charge, hydrophobicity, the possibility of forming biofilms, microbial concentration and exposure to the surrounding environment [1]. The severity of microbial infection occurs with forming biofilms.

Although antibiotic resistance evolves naturally via mutation, new resistance mechanisms are emerging and spreading globally, mainly due to the misuse and/or overuse of antibiotics, as well as improper infection prevention [9]. Antibiotic-resistant bacteria are considered as an emergent global disease and present a major public health care problem [29]. Studies made by European Centre for Disease Prevention and Control in 2016 showed that antimicrobial resistance remains a serious threat to public health in Europe [30]. Without urgent action, we are heading for a post-antibiotic era, in which common infections and minor injuries could lead to serious life-threatening conditions.

**Figure 4:**
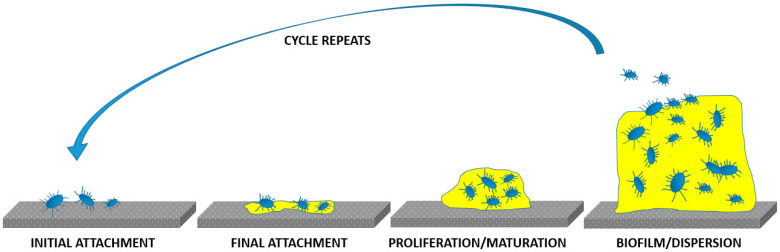
Schematic representation of biofilm formation on an implant’s surfaces.

SS implants have been associated with higher rates of infection than other metal implants; the material lacks inherent antibacterial properties and has a tendency to develop a fibrous fluid-filled capsules at the implant/surrounding tissues interface, which creates an ideal environment for bacterial colonization [31]. This can lead to formation of a biofilm that effectively shields the included microorganisms from immune cells and antibiotics. Biofilms prevent the intimate integration of an implant with surrounding tissues [32] and often lead to the development of chronic infections, leading to prolonged hospitalization, post-surgery complications, including implant-associated infections, implant rejection, and in extreme cases, death. The schematic representation of biofilm formation is presented in Figure 4. It involves several stages; initial attachment of microbes/adhesion to the implant, final attachment to the implant/microcolony and macrocolony formation due to maturation and biofilm dispersal [33].

In medical device-associated infections, pathogenic microorganisms take advantage of the foreign material and the impaired immune reaction, start to colonize the implant and form a biofilm. Biofilm formation is associated with antibiotic resistance in implant-associated infections [34]. Biofilm-embedded bacteria can easily adapt to the host defense mechanisms, which results in a decreased immune recognition and enhanced bacterial survival and persistence; implants create a niche for bacteria to evade the host defense by hiding in structural imperfections of the surface that are inaccessible for the larger immune cells, which ultimately results in bacterial persistence and chronicity of infection [35]. Conventional therapies that have been routinely used to control pathogens are being increasingly rendered ineffective. Current treatment concepts are thus based on the surgical removal of the infected tissue and strict antibiotic treatment to reduce bacterial burden as much as possible.

The use of SS as implant material is limited due to bacterial attachment and subsequent biofilm formation on its surface [36,37]. Biofilm is a self-produced polysaccharide matrix that adheres to the implant surface, protecting against bacterial degradation [38]. It is evident that surface morphology and roughness play a critical role in biofilm formation, hence surface modification is required [39,40,41,42]. It has been reported that by modifying surfaces to a micro/nano range, bacterial attachment is inhibited due to reduced contact between bacterial cell and the surface [43]. This is achieved by trapping the air in the substrate structure, in turn detaching the bacteria from its surface due to a reduced contact area. Also, bacteria can be trapped inside zones such as crevices, trenches or pits at the surface [44]. This biofilm formation depends on several factors such as the surface type, bacterial species and surface finishing mainly connected with the surface topography and chemistry. According to T.G. Slama, the antibacterial nature of Gram negative bacteria is attributed to its outer membrane surrounding the peptidoglycan layer [45]. This fluidic layer of Gram negative bacteria interacts differently with nanotextured surfaces as compared to a rigid peptidoglycan layer of Gram positive bacteria [46].

## 4. Common Strategies Used for Improving the Antimicrobial Properties of SS

### 4.1. Surface Chemistry

Scientists have been struggling to improve the efficacy of SS biomaterials’ bacterial activity by modifying the surface chemistry [47] through various antimicrobial agents [48] incorporated either in organic or in inorganic coatings [49,50,51]. While these approaches have generated improvements, they are complex and have a number of serious limitations. Specifically, antibiotic-loaded coatings on SS 316 are associated with the evolvement of multi-drug resistant microbes, while polymer coatings and their degradation products often contain harmful components and controlled release of substances is still hard to attain. Moreover, surface finishing methods, such as electro-polishing, have been described as evoking a local inflammatory response producing liquid-filled fibrous capsules at the SS 316 implant/bone surface that creates an ideal medium for bacterial colonization [52]. Fibrous capsules also prevent tissue integration with the implant and may reduce vascularization at the soft-tissue/implant interface. Such zones may induce rapid formation of neointimal hyperplasia and in-stent restenosis [53]. Fibrous capsule formation adjacent to an electro-polished SS implant is predominantly a result of its very smooth surface [54].

There are well known coatings based on ceramics and bioactive glasses that are quite biocompatible, but they lack the required strength for loading applications [53]. To provide the required strength, metal alloys can be coated with biochemical materials such as calcium phosphate or hydroxyapatite [53]. These bioceramic coatings increase the corrosion resistance of the implant surface on a metallic substrate. Hardystonite (Ca_2_ZnSi_2_O_7_) based ceramic induces biological fixation and tissue ingrowth at the tissue/implant interface, while the presence of zinc ions affects ceramic hardness. As reported by Ramaswami et al. [55], hardystonite ceramics support proliferation, grafting and differentiation of human osteoblast (HOB) cells and increase alkaline phosphatase activity. Ducheyne et al. have introduced an electrophoretic precipitation method (EPD) to cover hydroxyapatite (HA) on the surface of metal implants [56]. Improving the corrosion behavior of SS consequently enhances the biocompatibility of metallic implants in medicinal applications.

For improving corrosion resistance, Bagherpour et al. prepared hardystonite nano-bioceramic by using a sol gel method which was applied on SS 316L using an electrophoretic method [57]. SEM images confirm the formation of nanoparticles with small dimensions on a SS surface. Bio ceramic produced by the sol gel method forms a homogeneous and uniform coating. Also, the production of hardystonite nanoparticles along with the homogeneous coating is important for various applications. SEM images also revealed that by increasing the electrophoretic voltage hardystonite coating on SS 316L, it becomes more uniform. Uniformity of the layer depends on several factors, since increasing the voltage and coating time creates a thicker layer. Hardystonite coating reduces the corrosion current density of SS, contributing towards increased corrosion resistance of an implant, which in turn reduces its destructive effect on human body tissues.

The process of platinizing SS electrodes can be useful for modifying surfaces of short term implantable electrodes to have better charge transfer characteristics. The pH sensitivity of the hydrogel sensing interface can be improved by platinization of the sensing electrodes [58]. For this process, acid pickling of SS was reported by Aggas et al. [59]. Acid pickling of SS by immersing it in hydrochloric acid or sulfuric acid leads to removal of the chromium oxide layer due to the presence of chromium [60], and subsequent electrodeposition of platinum nanoparticles increases the resistance of SS to corrosion. Electrochemical impedance spectroscopy revealed that the electrodes prepared by this method show lower charge transfer resistance and passive layer resistance.

A method called the Laser Patterning Model [61] was used by Khalili and Sarafbidabad for surface modification of SS 304 by creating parallel micro grooves on the surface. This was followed by deposition of a thin fluorocarbon film using polytetrafluoroethylene (PTFE) sputtering. Wettability plays an eminent role in cellular connections and evaluation of effect on surface energy through various factors such as surface roughness, contact angle and oxygen content. Antonov et al. [62] suggest that microstructural changes of surface and oxygen content impact wettability. When a laser beam interacts with metal surface microstructural patterns in nanoscale that are obtained due to consecutive melting, this causes melt motion, evaporation and freezing on the surface [63]. High stability and hydrophobicity of the surface coating was obtained due to the presence of carbon fluorine links which are very electronegative. Also, the high consistency of these links reduce the contact of covalent bond with other molecules, which results in low surface energy followed by a decrease in friction, ultimately disposing water droplets and increasing the contact angle [64]. SEM images of laser treated SS reveal that a homogenous structure of microgrooves is obtained at the surface. Additionally, they reveal the deposition of a nanometer carbon fluorine coating forming micro grooves, which in turn develop a micro-nano hierarchical structure showing super hydrophobicity. A super hydrophobicity feature obtained on the SS surface can be utilized for different medical devices such as implants, stents and self-cleaning surfaces.

### 4.2. Nanostructure

Recent studies have shown a shift towards biomimetic/nano-structured surfaces, on which cell death is primarily caused by microbial membrane rupture via cellular adhesion [39]. Moreover, recently published studies claim that nanostructuring plays a pivotal role in the improvement of antimicrobial resistance. It was shown first in the case of titanium nanostructured surfaces that the adherence of oral bacteria can be modified by changes in nanostructured titanium surface properties such as nanotube diameter and/or fluoride content [65]. Later on, Wu et al. [66] demonstrated that nanoscale SS surface topographies also restrain bacterial adhesion and formation of microcolonies on nano-structured electro-polished surfaces. They studied the influence of electropolishing SS surfaces on bacterial adhesion and demonstrate that the nanoscale surface topographies inhibit bacterial adhesion and formation of microcolonies. Jang et al. [67] showed that SS 316L surfaces produced by electrochemical etching effectively inhibit bacterial adhesion of both Gram-negative *Escherichia coli* and Gram-positive *Staphylococcus aureus*. This was due to the formation of nanoprotrusions on the surface caused by electrochemical etching. When bacterial membrane is subjected to mechanical stress, it leads to membrane stretching, rupture and finally cell death.

Antibacterial effects of SS 316 were also improved by “nanocavitaton” [68]. This method is based on nanocavitating SS 304 and SS 316 surfaces via electrochemical anodization in a mixture of equal volumes of H_2_SO_4_ and aqueous H_2_O_2_. A distinctive surface nanotopography was obtained. This was due to the comprehensive dissociation and ionic conductivity, making the mixture suitable as an electrolyte. A mesoporous surface layer was produced with pore sizes of 16.4 ± 4.2 nm and 17.6 ± 7.1 nm for SS 304 and SS 316, respectively. The surface roughness (Ra) of the nanosurface was increased from 4.5 ± 0.8 nm before electrochemical treatment to 11.5 ± 2.7 nm (SS 304) and 9.7 ± 1.3 nm (SS 316) after treatment; this was revealed using Atomic force microscopy (AFM) images. Cell counts confirm that the nanostructured SS 304 and SS 316 facilitates the growth of both osteogenic cells and smooth muscle cells. More abundant filopodia of osteoblasts and smooth muscle cells (SMC) was observed on the surface of nanostructured SS through FE SEM images. Further investigation with HR TEM showed that filopodia established a close contact with nanosurfaces. When bacterial assay was performed, *E. coli* attached to the nanotextured surface of SS after 1 h of incubation and the situation remained unaltered after 4 h. This indicated the absence of significant proliferation of *E coli* on the SS surface. Co-culture of *E. coli* with osteoblast cell showed that the osteoblasts proliferated on the nanocavitated surface in the presence of bacteria. Furthermore, it was noticed that antibacterial activity was still retained on the modified surface.

**Figure 5:**
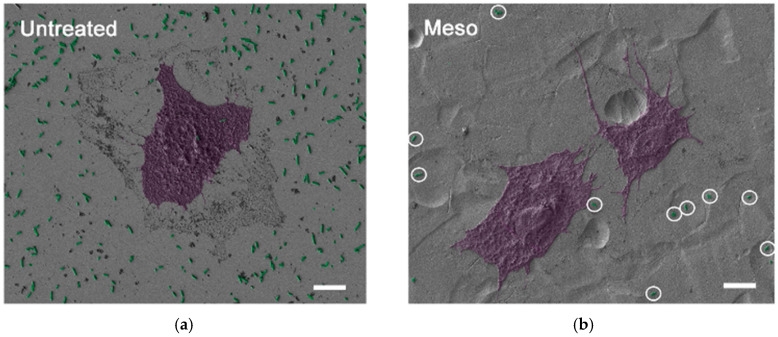
The adhesion of *E. coli* (marked green) and osteoblastic cells (marked purple) on the surface of (**a**) untreated SS and (**b**) electrochemically anodized SS, bar = 10 µm (adapted with permission from Ref. [68]).

To the best of our knowledge, a simple and cost-effective solution for helping to control bacterial infections of SS 316 has not been developed to date. However, recently (from 2018) a few promising studies claiming that nano-topographic effect of SS surfaces has an important impact on bacterial adhesion have emerged [66,68,69] (Figure 5). Further, previous investigations of other materials have presented biomimetic nanostructured surfaces as being antimicrobial [70], where cell death is supposedly caused by microbial membrane rupture via cellular adhesion [39]; however, a clear understanding of the underlying biocidal mechanism is still unknown. Surface roughened SS has been receiving attention due to its ability to induce advanced mechanical and physical characteristics on the treated surface through grain refinement, along with the effect of these characteristics on bacterial inhibition. Severe shot peening (SSP) [46] and magneto rheological abrasive flow finishing (MRAFF) [71] techniques have been reported for surface roughening. SSP treated SS showed enhanced mechanical properties and reduction in adhesion of Gram positive bacteria. In the case of MRAFF treated SS when surface roughness was minimum at nanometer level, a bacterial adhesion of 28 × 10^8^ CFU/mL was obtained for *Escherichia coli* with a roughness of 37.4 nm.

Lithography and photolithography moldings have been used to produce different micro/nano structures. Laser-induced periodic surface structures (LIPSS) on different surfaces is a novel surface modification technique offering new insights for numerous applications [46]. Among different methods, laser microstructuring has some advantages such as use of different environments, laser parameters and simplicity. The formation of femtosecond LIPSS on SS has been reported by several groups, revealing Low-spatial frequency LIPSS (LSFL) periods in the range of 500–700 nm [31,34]. LIPSS for nanostructuring is used for applications such as increasing tribological performance for contact mechanical applications, increasing surface roughness and causing ripple formation [72,73]. By using a laser on SS and varying laser parameters, two types of surface textures, namely nanospikes and nanoripples, are obtained [69]. The produced nanostructures vary for different grades of steel, while the nanoripple period is approximately 250 nm. Nanospiked surfaces exhibit hydrophillic properties depicting a good wetting behavior and for nanoripples, hydrophobicity is observed. In addition, the surface with nanospikes displays a better antibacterial effect compared to nanoripples. Approximately an 80% decrease in the bacterial population is observed for nanoripples through live/dead cells staining. Nickel (Ni) present in SS generates reactive oxygen species and ions, subsequently damaging the cellular structure. Various researchers [46,74,75,76] illustrate the toxicity of Ni to pathogenic microorganisms such as *S. aureus, Pseudomonas aeruginosa, Enterococcus faecalis* and *E. coli*. Ni nanoparticles can damage the DNA of bacteria when interacting with the phosphorus and sulfur contained inside them. Moreover, Ni nanoparticles generate hydrogen peroxide, causing bacterial cell death upon penetrating the bacteria [77].

Nune et al. [17] studied the effects of a nanograined/ultrafine grained (NG/UFG) structure alloyed with copper on the antimicrobial behavior of SS. The results indicated that a NG/UFG structure successfully inhibits biofilm formation as compared to its compact grained counterpart, and alloying with copper furthermore enhanced the effect [78]. Due to the high density of grain boundaries in nanocrystalline form, an increased diffusion path for Cr and Cu atoms to reach the surface and form a passive layer was achieved. That in turn hinders the interaction between surface and micro-organisms due to minimal free energy of the system [17].

Recent research on nano-textured surfaces, which mimic the bactericidal properties of plants, animals, insects and their topographical features has gained significant attention [38]. Due to the presence of hydrophobic structures and surface patterns of anti-biofouling surfaces like lotus leaves, taro leaves and shark skin, bacterial adhesion is inhibited. This shows that nanostructures with dense patterns improve the reduction rate of bacteria and particle attachment under water, compared to low density patterns [79,80]. Ivanova et al. showed that due to the presence of nanopillars on cicada wings, they can kill various kinds of bacteria, especially Gram negative cells [81,82]. Similarly Watson et al. found the same results on superhydrophobic Gecko skins [83]. Hasan et al. reports that by fabricating silicon nano-hairs using deep reactive ion etching, substrates can kill both Gram positive and negative cells [84]. Additionally Privett et al. suggest that by coating the surface of SS with fluorinated silica nanoparticles, biocidal effects against *S. aureus* and *P. aeruginosa* were observed [85]. This was observed due to factors such as surface free energy, surface charges and surface topography.

### 4.3. Wettability and Surface Energy

The material surface characteristics including appropriate surface roughness, high surface free-energy, low water contact angle, ultrafine grain structure and the appropriate chemical composition of the surface assist in increasing the antibacterial activity of the materials. Surfaces with unique surface characteristics such as wettability (superhydrophobicity and superhydrophilicity) [21] also exhibit microbial resistance through antifouling effect. Such surfaces form a barrier between bacteria and materials and prevent direct contact. When the surface energy of bacterial cells is smaller than the surface energy of liquids in which cells are suspended, cells preferentially attach to hydrophobic materials. The mode of action of superhydrophobic surfaces to reduce bacterial adhesion is, however, still not clear, mainly due to other surface parameters (like nanotopography) which may synergistically influence biological responses. The antibacterial effect of nanotextured surfaces has been attributed to repulsive forces that are increased by the relatively large surface area of nanotopographies [1]. However, Rodriguez-Contreras et al. [68] report that it is likely that topography itself may account for the poor ability of bacteria to adhere to nanocavities formed on the surface of SS rather than the marginally altered wettability of the material after electrochemical anodization [21].

## 5. Use of Plasma Technologies for Antimicrobial Properties of SS

Gaseous plasma surface treatment changes surface chemistry composition, which can be tailored according to specific needs (using different gases for example). Plasma may also affect the surface morphology, increasing roughness on a nano-scale, again in a controlled manner (by plasma power or plasma treatment time). The combination of both, chemical and physical modifications increases free surface energy of plasma treated surface, causing the change of surface wettability. By tailoring gases and plasma parameters, wettability can either increase or decrease. Moreover, plasma modification only alters the top surface layer of material and keeps the bulk attributes of the material intact.

Plasma in this context is the fourth state of matter, defined as excited, highly reactive, electro-conductive gas. It consists of a variety of charged particles with the ability to change surface characteristics of numerous materials including SS. As described previously, SS has all of the desired mechanical characteristics and is considered as a superior material for medical applications such as medical tools, hospital surfaces, implants etc. There is only one major drawback, which is its biological inertness [86]. It has practically no antibacterial effect.

SS materials are on one hand used as surfaces in hospitals which are bound to get infected and sooner or later transmit bacterial infections, posing a huge health risk. Statistically, hospital acquired infections (HAIs) are ranked in the top ten leading causes of death in the USA [87], and 5000 patients out of 300,000 infected by HAIs die in the UK [88]. On the other hand, SS is vastly employed for permanent or temporary implants, where its biologically inert properties do not provide sufficient growth and proliferation of host cells over bacteria. This presents a serious risk of infections taking into consideration that immune systems are highly impaired after implant surgery and that bacterial infections are common [86].

All of the above mentioned issues lead researchers to implement different plasma techniques in order to improve the antibacterial surface characteristics of SS without changing its desirable bulk characteristics. The prevailing idea found in literature is to improve SS surfaces by coating them with silver (Ag) or copper (Cu) ion doped coatings, due to their excellent non-specific antibacterial characteristics [89,90,91]. In the case of hospital surfaces, coatings must be thick enough to withstand everyday use and preferably to last for years. Conventional physical vapor deposition (PVD) techniques, such as plasma ion implantation (PII), and plasma enhanced chemical vapor deposition (PE-CVD) techniques for example, produce coating layers up to 1 µm thick, which is not enough for repeated use [89,92]. Research groups report different plasma setups and combinations of various techniques to overcome this issue.

Zhang et al. [89] used a plasma surface alloying technique and successfully deposited a 26 µm thick layer of copper onto SS 304 and achieved a 100% reduction of *E. coli* and *S. aureus* in 3 h of exposure time. Miola et al. [86] reported plasma sprayed glass coatings doped with silver where the reached thicknesses were between 70 and 150 µm, which exhibited bacteriostatic behavior proportional to the amount of silver introduced. Double glow plasma silvering in combination with active screen plasma nitriding was proposed by Dong et al. [92], who claim that coating thickness between 10 and 300 µm can be controlled by means of changing plasma discharge parameters.

Implants, on the other hand, impose another kind of challenge. Surface silver doping was preferred for this application; however, the long lasting antibacterial effect is obtained only if an appropriate amount of silver is released, but it should also be considered that silver at a certain concentration becomes toxic. For example, Miola et al. [86] report the rapid release of silver immersed in simulated body fluid (SBF), where all of the silver was released within 24 h. This suggests that the right amount of silver could help prevent bacterial infection after implantation surgery and dissolve afterwards. Additionally, Baba et al. [93] suggest plasma ion implantation with magnetron sputtering for the production of diamond like carbon (DLC) films containing silver, which are hemocompatible and antibacterial, and are capable of reducing the *S. aureus* by 80% after 6 h of incubation time, even with the smallest concentrations of silver (3.8 at.%).

Apart from low pressure techniques where plasma is mostly used for indirect treatment of surfaces, novel atmospheric pressure plasma techniques for direct surface treatment are gaining importance in the field of antibacterial coatings [94,95]. Ibis et al. [95] reported on SS 304 and 316L treated with dielectric barrier discharge (DBD) plasma configuration in air and tested for antibacterial properties against *E. coli* and *S. aureus*. After 10 min of plasma treatment, 4-log inactivation of both *E. coli* and *S. aureus* was observed on SS 304 and 316L.

In our recent study, presented below, low pressure, radio frequency (RF) and inductively coupled plasma were used for modification of the surface properties of SS 316L by RF plasma was ignited inside a glass reactor and used for modification of SS 316L samples with oxygen, hydrogen and nitrogen as gas carriers. Different treatment conditions like treatment time and power output were also were used and obtained results indicate that altered surface chemistry, morphology and wettability can be obtained, which significantly influences the biological response. A short summary of the important findings concerning surface features obtained from Atomic force microscopy (AFM), profilometry, Secondary ion mass spectrometry (SIMS), X-ray photoelectron spectroscopy (XPS) and water contact angle (WCA) analyses are presented herein.

**Figure 6:**
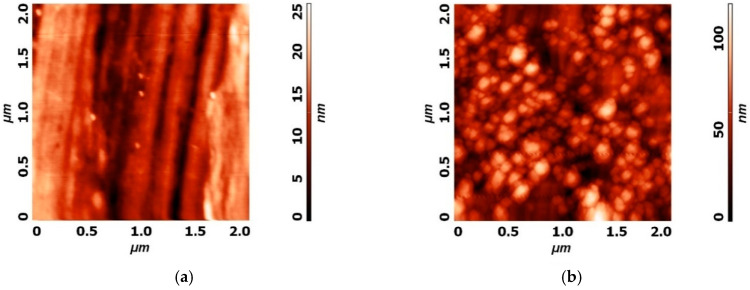
Atomic force microscopy (AFM) image of (**a**) 2 µm × 2 µm area of untreated SS 316L, (**b**) 2 µm × 2 µm area of plasma treated SS 316L with a high amount of CrO on the surface.

AFM analysis of untreated plasma modified surfaces was performed. It was found that ripples occur after plasma treatment on the previously rather smooth surface of SS. Plasma physical and chemical etching is a well-known phenomenon responsible for this transformation. As can be seen from Figure 6, small ripples between 50 and 100 nm are present after two stage plasma treatment, starting with 60 s of H-mode hydrogen plasma at the pressure of 20 Pa (base pressure 3 Pa) and power of 700 W and the addition of oxygen in the second step, lasting for 20 s at a combined pressure of 30 Pa (3 Pa base pressure, up to 20 Pa hydrogen, up to 30 Pa oxygen).

**Figure 7:**
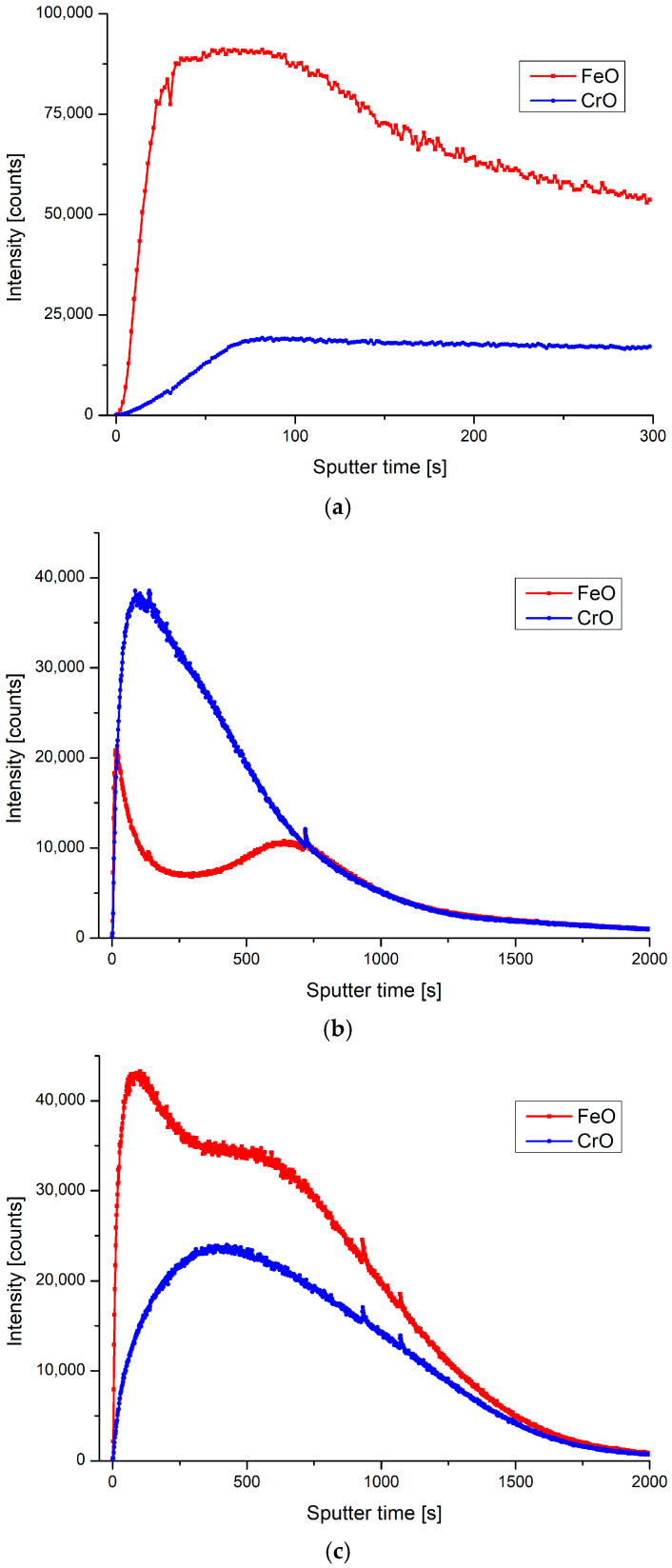
SIMS analysis of SS 316L, (**a**) untreated, (**b**) plasma treated with prevailing CrO, (**c**) plasma treated with prevailing FeO.

**Figure 8:**
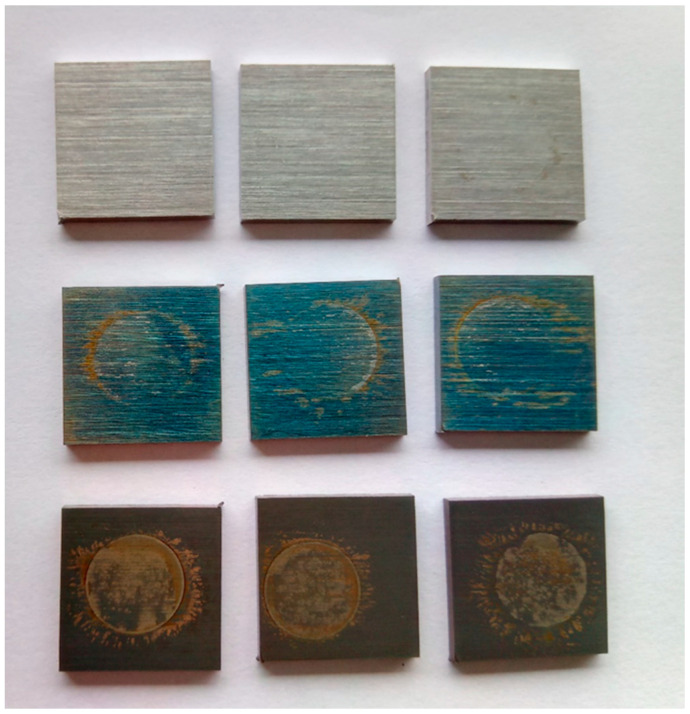
Several 20 mm × 20 mm × 2 mm samples after corrosion analysis, top-bottom; untreated, Plasma 1—CrO, Plasma 2—FeO.

The aforementioned plasma treatment also altered the surface color from silver to metallic blue, and an in-depth SIMS profile revealed that top surface layer is prevailingly covered by CrO, as shown in Figure 7b. Although CrO is already found on SS 316L surface in an untreated state (Figure 7a), the thickness of the layer seems to increase after this type of plasma exposure. While the sample was treated at the same conditions, only increasing the treatment time in the second step from 20 s to 80 s, an increase in FeO was observed. According to SIMS analysis, the FeO peak was moved and increased, as seen in Figure 7c. The reason for this is probably due to an increase in the sample temperature because of a longer bombardment with highly reactive oxygen species. In this case, the sample color was dark brown and had no shine, as can be seen from Figure 8.

Profilometry measurements were also conducted and average roughness Ra (nm) was calculated from 3 separate measurements. The single measurement length was 4.1 mm, perpendicular to the surface razes from grinding, as seen in Figure 8. Results on average surface roughness including standard deviations are presented in Table 1 below. It can be observed that on both plasmas increased SS surfaces Ra, due to nanostructuring of the surfaces, as shown in Figure 6.

The surface roughness is undoubtedly one of the main factors [7,19], influencing attachment of proteins, cells and bacteria to the surfaces as well as biofilm formation. It is believed that a properly modified surface can prevent bacteria from attaching or even cause membrane stretching and cell death [67]. An increase in surface roughness was observed for both plasma treated samples.

**Figure 9:**
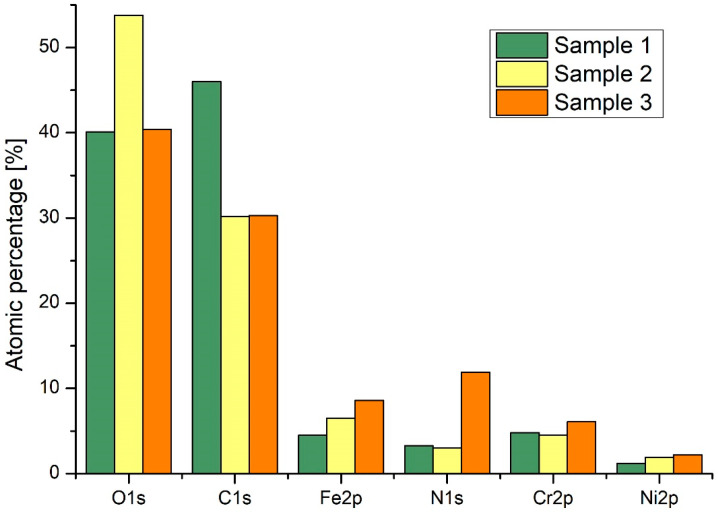
XPS results on SS 316L, sample 1 is untreated as received, sample 2 and 3 plasma were treated using oxygen and nitrogen, respectively (plasma conditions; 40 Pa, RF 13.56 MHz, 700 W H-mode, 1 s).

The effect of different plasma modification of SS 316L on chemical composition was studied by using XPS. The same plasma setup was used, while different gases were used for modification. Treatment was done for 1 s at 40 Pa pressure and a power level of 700 W using oxygen or nitrogen carrier gas. The chemical surface compositions are compared in Figure 9. An untreated sample of SS 316L was also analyzed, in an “as received” state (passivated surface).

Corrosion resistance can be interpreted by stability of a passive film on the surface deeply connected with surface chemistry. An increase of the reliability of SSs can be achieved with passive film stability. In the case of NO gas, nitrogen has been presented as an amplifier of corrosion resistance. Nitrogen is known to widen the passive range and decrease passive current density, which is governing to a positive resistance effects against cracking and intergranular corrosion. There is some speculation on the incorporation of nitrogen into the passive film, a formation of nitrate ions with repulsive action against Cl^−^ and either with the formation of NH4^+^ ions, resulting in repassivation [96].

In case of biological responses on enhanced corrosion on biomaterials, a release of metal ions on the surface affects cell adhesion (osseointegration), especially cells osteocytes, contributing to an aseptic loss of the implant [97]. With plasma treatment, the surface elemental chemistry is modified to improve biomechanical properties for successful implantation [98].

Surface charge and polarity, induced by plasma treatment, can influence antibacterial properties. A strong surface charge can repel or attract bacteria by itself, preventing the colonization and formation of biofilm, mediated by proteins and/or other particles with distinctive internal charge distribution [7,19].

**Figure 10:**
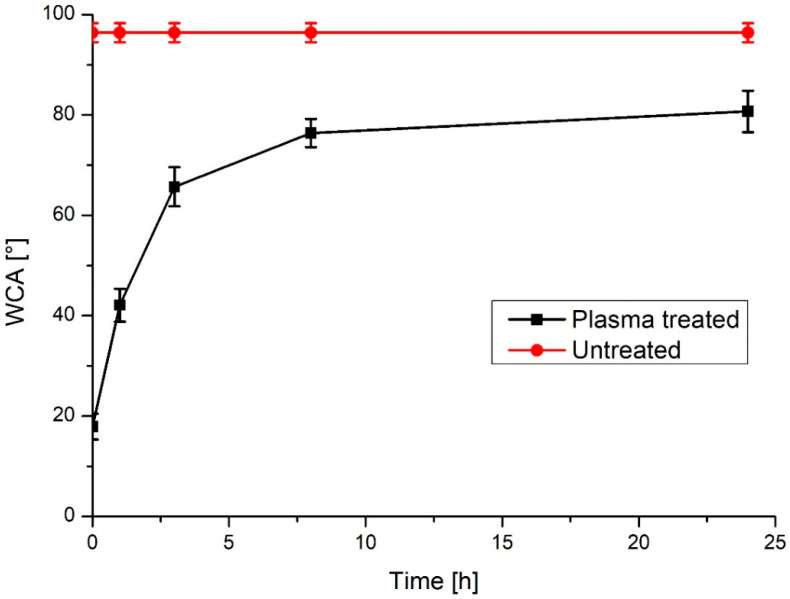
Ageing of plasma treatment effects, immediately after plasma treatment, after 1, 3, 8 and 24 h. A line for the WCA (96,4°) of untreated sample is available for comparison.

Water contact angle (WCA) is one of the fastest methods for analyzing the effects of plasma treatment on a surface charge and free energy. By measuring WCA through a period of time, ageing effects of plasma treatment [20,67] can be monitored. These effects vary greatly between different materials and are also strongly dependent on plasma parameters. In case of SS, we found that ageing starts immediately after plasma treatment and reaches final value of WCA, which is lower than untreated sample, one day after plasma treatment (Figure 10). This suggests that ageing of plasma treated surfaces should be considered and if improved wettability is desired for specific biological response (osteoblast adhesion, antibacterial effects), plasma treatment should be conducted on sight before use of SS material for specific application. In the case of orthopedic implants plasma treatment prior to implementation will provide for improved proliferation of osteoblast cells, which will reduce the risk of bacterial adhesion, as growth of cells and proteins covering the implant surface will occur.

## 6. Conclusions

In this review article, the use of SS as biomaterial was presented and strategies used to alter its surface properties were discussed. It was shown that extensive studies on surface modification of SS exist and that there are still many ongoing studies on this topic. This is mainly due to the vast use of SS in medical applications, as well as due to an increase in antibiotic resistant bacteria strains. Although SS is one of the most frequently used biomaterials, with excellent mechanical properties, it still lacks desired biofunctionality and antibacterial properties. So far, different approaches have been employed in order to prevent bacterial adhesion and biofilm formation on SS surfaces. Mainly the aim was to alter surface chemistry, topography (nanotopography) and wettability or surface energy. The most common approaches are based on the coating of surfaces with various types of organic or inorganic coatings which may include controlled release of substances like Ag, Zn, SiO_2_ or even drug release (antibiotic). In the case of coatings based on antibiotic release, the connection with multi-drug resistant microbes was observed and it should be emphasized that in many cases, the desired amount of substance is hard to attain in the real biological environment. On the other hand, polymer based coatings are also frequently used, however the main concern in this case is its stability, as degradation products which contain harmful components may be released, while in the case of friction (like hip implants), the release of small particles which can evoke cytotoxic reactions occur. Other methods dealing with electro-polishing are also common, however they were correlated by evoking a local inflammatory response on a SS 316 implant. Alteration of surface morphology (microstructure and nanostructure) was employed in recent years as one of the prospective approaches for prevention of bacterial adhesion. For example, lithography and photolithography moldings were used to produce different micro/nano structures, while for fabrication of specific nanotopography, many approaches were based on electrochemical anodization techniques, which enabled formation of the desired nanostructure. Although many of the approaches indeed resulted in a reduction or even prevention of bacterial adhesion, their drawback was mainly connected with insufficient mechanical stability of the final coating or/and timely and high cost of the proposed approach. However, in many cases the main goal was to study and gain knowledge on currently not well known mechanisms of bacterial adhesion. Approaches based on plasma technologies [99] which include already commercially available techniques such as plasma vapor deposition (PVD) or plasma electrolytic oxidation (PEO), as well as novel approaches presented herein, show high potential as they enable rapid surface modification, are environmentally friendly and can be easily implemented into manufacturing processes. However, their efficiency in obtaining antimicrobial properties of surfaces is sometimes still not satisfactory.

Many different approaches have been attempted to thwart microbial corrosion [100], to counteract bacterial adhesion [101] or to confer bactericidal or antibacterial properties to the material surfaces [102,103], while also improving biological responses, such as hemocompatibility [101,103]. Future research will undoubtedly be aimed at optimizing the biological performances of materials, such as SS, by modifying their surfaces. In this short review, the influence of various treatment techniques was presented and discussed. It was shown that there is still a limited amount of available treatment approaches that enable desired biological responses of SS, especially under in vivo conditions. Thus, novel approaches are still sought. Further research with an emphasis on nanostructured or even biomimetic surfaces on this topic is foreseen, as it presents a new unique way to obtain desired biological responses.

## Figures and Tables

**Table 1:** Average surface roughness Ra (nm) of SS 316L samples with standard deviations from 3 measurements.

Sample	Ra (nm)	Stdandard Deviation (nm)
Untreated	132	6
Plasma 1 (CrO)	157	8
Plasma 2 (FeO)	146	4

## References

[1] Bakterij A.J.M.T. (2014). An overview of the influence of stainless-steel surface properties on bacterial adhesion. Mater. Tehnol..

[2] Hermawan H., Ramdan D., Djuansjah J.R. (2011). Metals for biomedical applications. Biomed. Eng. Theory Appl..

[3] Beaupré G.S., Csongradi J.J. (1996). Refracture risk after plate removal in the forearm. J. Orthop. Trauma.

[4] Boyd A.H., Hylwa S.A. (2018). Nickel release from surgical instruments and operating room equipment. Dermatol. Online J..

[5] Neupane M.P., Park I.S., Lee S.J., Kim K.A., Lee M.H., Bae T.S. (2009). Study of anodic oxide films of titanium fabricated by voltammetric technique in phosphate buffer media. Int. J. Electrochem. Sci.

[6] Orapiriyakul W., Young P.S., Damiati L., Tsimbouri P.M. (2018). Antibacterial surface modification of titanium implants in orthopaedics. Journal of tissue engineering.

[7] Kulkarni M., Mazare A., Gongadze E., Perutkova Š., Kralj-Iglič V., Milošev I., Schmuki P., Iglič A., Mozetič M. (2015). Titanium nanostructures for biomedical applications. Nanotechnology.

[8] Santos M., Filipe E.C., Michael P.L., Hung J., Wise S.G., Bilek M.M. (2016). Mechanically robust plasma-activated interfaces optimized for vascular stent applications. ACS Appl. Mater. Interfaces.

[9] Benčina M., Mavrič T., Junkar I., Bajt A., Krajnović A., Lakota K., Žigon P., Sodin-Šemrl S., Kralj-Iglič V., Iglič A. (2018). The Importance of Antibacterial Surfaces in Biomedical Applications. Advances in Biomembranes and Lipid Self-Assembly.

[10] Milosev L., Antolic V., Minovic A., Cor A., Herman S., Pavlovcic V., Campbell P. (2000). Extensive metallosis and necrosis in failed prostheses with cemented titanium-alloy stems and ceramic heads. J. Bone Jt. Surg. Br. Vol..

[11] Villapún V.M., Dover L.G., Cross A., González S. (2016). Antibacterial metallic touch surfaces. Materials.

[12] Różańska A., Chmielarczyk A., Romaniszyn D., Sroka-Oleksiak A., Bulanda M., Walkowicz M., Osuch P., Knych T. (2017). Antimicrobial properties of selected copper alloys on Staphylococcus Aureus and Escherichia Coli in different simulations of environmental conditions: With vs. without organic contamination. Int. J. Environ. Res. Public Health.

[13] Junkar I., Kulkarni M., Drašler B., Rugelj N., Mazare A., Flašker A., Drobne D., Humpolíček P., Resnik M., Schmuki P. (2016). Influence of various sterilization procedures on TiO_2_ nanotubes used for biomedical devices. Bioelectrochemistry.

[14] Kopf B.S., Schipanski A., Rottmar M., Berner S., Maniura-Weber K. (2015). Enhanced differentiation of human osteoblasts on Ti surfaces pre-treated with human whole blood. Acta Biomater..

[15] Stathopoulos P., Theodossiades G., Mourouzis C., Evangelou A. (2011). Effect of titanium maxillofacial implants and osteosynthesis materials on platelet function. Br. J. Oral Maxillofac. Surg..

[16] Gibon E., Amanatullah D.F., Loi F., Pajarinen J., Nabeshima A., Yao Z., Hamadouche M., Goodman S.B. (2017). The biological response to orthopaedic implants for joint replacement: Part I: Metals. J. Biomed. Mater. Res. Part B Appl. Biomater..

[17] Nune K., Somani M., Spencer C., Misra R. (2017). Cellular response of Staphylococcus aureus to nanostructured metallic biomedical devices: Surface binding and mechanism of disruption of colonization. Mater. Technol..

[18] Harris L.G., Meredith D.O., Eschbach L., Richards R.G. (2007). Staphylococcus aureus adhesion to standard micro-rough and electropolished implant materials. J. Mater. Sci. Mater. Med..

[19] Gongadze E., Kabaso D., Bauer S., Slivnik T., Schmuki P., Van Rienen U., Iglič A. (2011). Adhesion of osteoblasts to a nanorough titanium implant surface. Int. J. Nanomed..

[20] Junkar I., Kulkarni M., Benčina M., Kovač J., Mrak-Poljšak K.a., Lakota K., Sodin-Šemrl S.n., Mozetič M., Iglič A. (2020). Titanium Dioxide Nanotube Arrays for Cardiovascular Stent Applications. ACS Omega.

[21] Kulkarni M., Patil-Sen Y., Junkar I., Kulkarni C.V., Lorenzetti M., Iglič A. (2015). Wettability studies of topologically distinct titanium surfaces. Colloids Surf. B Biointerfaces.

[22] Kulkarni M., Mazare A., Park J., Gongadze E., Killian M.S., Kralj S., von der Mark K., Iglič A., Schmuki P. (2016). Protein interactions with layers of TiO_2_ nanotube and nanopore arrays: Morphology and surface charge influence. Acta Biomater..

[23] Zhao J., Zhai Z., Sun D., Yang C., Zhang X., Huang N., Jiang X., Yang K. (2019). Antibacterial durability and biocompatibility of antibacterial-passivated 316L stainless steel in simulated physiological environment. Mater. Sci. Eng. C.

[24] Jana S. (1992). Effect of heat input on the HAZ properties of two duplex stainless steels. J. Mater. Process. Technol..

[25] Sedriks A.J. (1996). Corrosion of Stainless Steel.

[26] Tanzi M.C., Farè S., Candiani G. (2019). Foundations of Biomaterials Engineering.

[27] Manam N., Harun W., Shri D., Ghani S., Kurniawan T., Ismail M.H., Ibrahim M. (2017). Study of corrosion in biocompatible metals for implants: A review. J. Alloys Compd..

[28] Ojeil M., Jermann C., Holah J., Denyer S.P., Maillard J.-Y. (2013). Evaluation of new in vitro efficacy test for antimicrobial surface activity reflecting UK hospital conditions. J. Hosp. Infect..

[29] Roca I., Akova M., Baquero F., Carlet J., Cavaleri M., Coenen S., Cohen J., Findlay D., Gyssens I., Heure O.E. (2015). The global threat of antimicrobial resistance: Science for intervention. New Microbes New Infect..

[30] Summary of the latest data on antibiotic resistance in the European Union 2017. https://www.ecdc.europa.eu/sites/default/files/documents/EAAD%20EARS-Net%20summary.pdf.

[31] Chen J., Howell C., Haller C.A., Patel M.S., Ayala P., Moravec K.A., Dai E., Liu L., Sotiri I., Aizenberg M. (2017). An immobilized liquid interface prevents device associated bacterial infection in vivo. Biomaterials.

[32] Park C., Lee S.-W., Kim J., Song E.-H., Jung H.-D., Park J.-U., Kim H.-E., Kim S., Jang T.-S. (2019). Reduced fibrous capsule formation at nano-engineered silicone surfaces via tantalum ion implantation. Biomater. Sci..

[33] Arciola C.R., Campoccia D., Montanaro L. (2018). Implant infections: Adhesion, biofilm formation and immune evasion. Nat. Rev. Microbiol..

[34] Li B., Webster T.J. (2018). Bacteria antibiotic resistance: New challenges and opportunities for implant-associated orthopedic infections. J. Orthop. Res..

[35] Seebach E., Kubatzky K.F. (2019). Chronic implant-related bone infections–can immune modulation be a therapeutic strategy?. Front. Immunol..

[36] Hsu L.C., Fang J., Borca-Tasciuc D.A., Worobo R.W., Moraru C.I. (2013). Effect of micro-and nanoscale topography on the adhesion of bacterial cells to solid surfaces. Appl. Environ. Microbiol..

[37] Whitehead K.A., Colligon J., Verran J. (2005). Retention of microbial cells in substratum surface features of micrometer and sub-micrometer dimensions. Colloids Surf. B Biointerfaces.

[38] Ferraris S., Spriano S. (2016). Antibacterial titanium surfaces for medical implants. Mater. Sci. Eng. C.

[39] Jaggessar A., Shahali H., Mathew A., Yarlagadda P.K. (2017). Bio-mimicking nano and micro-structured surface fabrication for antibacterial properties in medical implants. J. Nanobiotechnol..

[40] Saraeva I.N., Tolordava E.R., Nastulyavichus A.A., Ivanova A.K., Kudryashov S.I., Rudenko A.A., Melnik N.N., Zayarny D.A., Ionin A.A., Romanova Y.M. (2020). A bacterial misericorde: Laser-generated silicon nanorazors with embedded biotoxic nanoparticles combat the formation of durable biofilms. Laser Phys. Lett..

[41] Krylach I., Kudryashov S., Olekhnovich R., Moskvin M., Uspenskaya M. (2019). Tuning water wetting angle of a steel surface via nanosecond laser ablative nano/microtexturing for chemical and biomedical microfluidic applications. Laser Phys. Lett..

[42] Saltuganov P., Ionin A., Kudryashov S., Rukhadze A., Gavrilov A., Makarov S., Rudenko A., Zayarny D. (2015). Fabrication of superhydrophobic coating on stainless steel surface by femtosecond laser texturing and chemisorption of an hydrophobic agent. J. Russ. Laser Res..

[43] Thomas V., Dean D.R., Vohra Y.K. (2006). Nanostructured biomaterials for regenerative medicine. Curr. Nanosci..

[44] Kargar M., Wang J., Nain A.S., Behkam B. (2012). Controlling bacterial adhesion to surfaces using topographical cues: A study of the interaction of Pseudomonas aeruginosa with nanofiber-textured surfaces. Soft Matter.

[45] Slama T.G. (2008). Gram-negative antibiotic resistance: There is a price to pay. Crit. Care.

[46] Bagherifard S., Hickey D.J., de Luca A.C., Malheiro V.N., Markaki A.E., Guagliano M., Webster T.J. (2015). The influence of nanostructured features on bacterial adhesion and bone cell functions on severely shot peened 316L stainless steel. Biomaterials.

[47] Yang W.J., Cai T., Neoh K.-G., Kang E.-T., Teo S.L.-M., Rittschof D. (2013). Barnacle cement as surface anchor for “clicking” of antifouling and antimicrobial polymer brushes on stainless steel. Biomacromolecules.

[48] Cécius M., Jérôme C. (2011). A fully aqueous sustainable process for strongly adhering antimicrobial coatings on stainless steel. Prog. Org. Coat..

[49] Liu Y., Zheng Z., Zara J.N., Hsu C., Soofer D.E., Lee K.S., Siu R.K., Miller L.S., Zhang X., Carpenter D. (2012). The antimicrobial and osteoinductive properties of silver nanoparticle/poly (DL-lactic-co-glycolic acid)-coated stainless steel. Biomaterials.

[50] Pradhan D. (2016). Sol-Gel Prepared Niobium Oxide and Silicon Oxide Coatings on 316L Stainless Steel for Biomedical Applications. Ph.D. Thesis.

[51] Ciacotich N., Din R.U., Sloth J.J., Møller P., Gram L. (2018). An electroplated copper–silver alloy as antibacterial coating on stainless steel. Surf. Coat. Technol..

[52] Hayes J.S., Richards R.G. (2010). Surfaces to control tissue adhesion for osteosynthesis with metal implants: In vitro and in vivo studies to bring solutions to the patient. Expert Rev. Med. Devices.

[53] Koniari I., Kounis N.G., Hahalis G. (2016). In-stent restenosis and thrombosis due to metal hypersensitivity: Implications for Kounis syndrome. J. Thorac. Dis..

[54] Hayes J., Klöppel H., Wieling R., Sprecher C., Richards R. (2018). Influence of steel implant surface microtopography on soft and hard tissue integration. J. Biomed. Mater. Res. Part B Appl. Biomater..

[55] Ramaswamy Y., Wu C., Zhou H., Zreiqat H. (2008). Biological response of human bone cells to zinc-modified Ca–Si-based ceramics. Acta Biomater..

[56] Ducheyne P., Van Raemdonck W., Heughebaert J., Heughebaert M. (1986). Structural analysis of hydroxyapatite coatings on titanium. Biomaterials.

[57] Bagherpour I. Fabrication of hardystonite nano-bioceramic coating on 306L stainless steel substrate using electrophoretic method and evaluation of its corrosion resistance to improve medical performance. Proceedings of the TMS 2019 148th Annual Meeting & Exhibition Supplemental Proceedings; Henry B. González Convention Center.

[58] Bhat A., Smith B., Dinu C.-Z., Guiseppi-Elie A. (2019). Molecular engineering of poly (HEMA-co-PEGMA)-based hydrogels: Role of minor AEMA and DMAEMA inclusion. Mater. Sci. Eng. C.

[59] Aggas J.R., Bhat A., Walther B.K., Guiseppi-Elie A. (2019). Nano-Pt ennobling of stainless steel for biomedical applications. Electrochim. Acta.

[60] Cowley A. (2011). A healthy future: Platinum in medical applications. Platin. Met. Rev..

[61] Khalili E., Sarafbidabad M. (2017). Combination of laser patterning and nano PTFE sputtering for the creation a super-hydrophobic surface on 304 stainless steel in medical applications. Surf. Interfaces.

[62] Antonov E., Bagratashvili V., Popov V., Ball M., Grant D., Howdle S., Scotchford C. (2003). Properties of calcium phosphate coatings deposited and modified with lasers. J. Mater. Sci. Mater. Med..

[63] Kurella A., Dahotre N.B. (2005). Surface modification for bioimplants: The role of laser surface engineering. J. Biomater. Appl..

[64] Hao L., Lawrence J. (2006). Albumin and fibronectin protein adsorption on CO_2_-laser-modified biograde stainless steel. Proc. Inst. Mech. Eng. Part H J. Eng. Med..

[65] Narendrakumar K., Kulkarni M., Addison O., Mazare A., Junkar I., Schmuki P., Sammons R., Iglič A. (2015). Adherence of oral streptococci to nanostructured titanium surfaces. Dental Mater..

[66] Wu S., Altenried S., Zogg A., Zuber F., Maniura-Weber K., Ren Q. (2018). Role of the surface nanoscale roughness of stainless steel on bacterial adhesion and microcolony formation. ACS Omega.

[67] Jang Y., Choi W.T., Johnson C.T., García A.J., Singh P.M., Breedveld V., Hess D.W., Champion J.A. (2017). Inhibition of bacterial adhesion on nanotextured stainless steel 316L by electrochemical etching. ACS Biomater. Sci. Eng..

[68] Rodriguez-Contreras A., Bello D.G., Flynn S., Variola F., Wuest J.D., Nanci A. (2018). Chemical nanocavitation of surfaces to enhance the utility of stainless steel as a medical material. Colloids Surf. B Biointerfaces.

[69] Nastulyavichus A., Kudryashov S., Saraeva I., Smirnov N., Rudenko A., Tolordava E., Zayarny D., Gonchukov S., Ionin A. (2019). Nanostructured steel for antibacterial applications. Laser Phys. Lett..

[70] Elbourne A., Crawford R.J., Ivanova E.P. (2017). Nano-structured antimicrobial surfaces: From nature to synthetic analogues. J. Colloid Interface Sci..

[71] Kathiresan S., Mohan B. (2017). In-vitro bacterial adhesion study on stainless steel 316L subjected to magneto rheological abrasive flow finishing. Biomed. Res..

[72] Derrien T.-Y., Torres R., Sarnet T., Sentis M., Itina T.E. (2012). Formation of femtosecond laser induced surface structures on silicon: Insights from numerical modeling and single pulse experiments. Appl. Surf. Sci..

[73] Eichstädt J., Römer G., Huis A. (2011). Towards friction control using laser-induced periodic surface structures. Phys. Procedia.

[74] Zhang W., Li Y., Niu J., Chen Y. (2013). Photogeneration of reactive oxygen species on uncoated silver, gold, nickel, and silicon nanoparticles and their antibacterial effects. Langmuir.

[75] Kumar H., Rani R., Salar R. (2010). Reverse micellar synthesis, characterization & antibacterial study of nickel nanoparticles. Adv. Control Chem. Eng. Civil Eng. Mech. Eng..

[76] Chaudhary R.G., Tanna J.A., Gandhare N.V., Rai A.R., Juneja H.D. (2015). Synthesis of nickel nanoparticles: Microscopic investigation, an efficient catalyst and effective antibacterial activity. Adv. Mater. Lett.

[77] Kokkoris M., Trapalis C., Kossionides S., Vlastou R., Nsouli B., Grötzschel R., Spartalis S., Kordas G., Paradellis T. (2002). RBS and HIRBS studies of nanostructured AgSiO_2_ sol–gel thin coatings. Nucl. Instrum. Methods Phys. Res. Sect. B Beam Interact. Mater. Atoms.

[78] Yu B., Lesiuk A., Davis E., Irvin R.T., Li D. (2010). Surface nanocrystallization for bacterial control. Langmuir.

[79] Tian P., Guo Z. (2017). Bioinspired silica-based superhydrophobic materials. Appl. Surf. Sci..

[80] Reiner Ž., Catapano A.L., De Backer G., Graham I., Taskinen M.-R., Wiklund O., Agewall S., Alegria E., Chapman M.J., Durrington P. (2011). ESC/EAS Guidelines for the management of dyslipidaemias: The Task Force for the management of dyslipidaemias of the European Society of Cardiology (ESC) and the European Atherosclerosis Society (EAS). Eur. Heart J..

[81] Ivanova E.P., Hasan J., Webb H.K., Truong V.K., Watson G.S., Watson J.A., Baulin V.A., Pogodin S., Wang J.Y., Tobin M.J. (2012). Natural bactericidal surfaces: Mechanical rupture of Pseudomonas aeruginosa cells by cicada wings. Small.

[82] Hasan J., Webb H.K., Truong V.K., Pogodin S., Baulin V.A., Watson G.S., Watson J.A., Crawford R.J., Ivanova E.P. (2013). Selective bactericidal activity of nanopatterned superhydrophobic cicada Psaltoda claripennis wing surfaces. Appl. Microbiol. Biotechnol..

[83] Watson G.S., Green D.W., Schwarzkopf L., Li X., Cribb B.W., Myhra S., Watson J.A. (2015). A gecko skin micro/nano structure–A low adhesion, superhydrophobic, anti-wetting, self-cleaning, biocompatible, antibacterial surface. Acta Biomater..

[84] Hasan J., Raj S., Yadav L., Chatterjee K. (2015). Engineering a nanostructured “super surface” with superhydrophobic and superkilling properties. RSC Adv..

[85] Privett B.J., Youn J., Hong S.A., Lee J., Han J., Shin J.H., Schoenfisch M.H. (2011). Antibacterial fluorinated silica colloid superhydrophobic surfaces. Langmuir.

[86] Miola M., Ferraris S., Di Nunzio S., Robotti P., Bianchi G., Fucale G., Maina G., Cannas M., Gatti S., Massé A. (2009). Surface silver-doping of biocompatible glasses to induce antibacterial properties. Part II: Plasma sprayed glass-coatings. J. Mater. Sci. Mater. Med..

[87] Emori T.G., Banerjee S.N., Culver D.H., Gaynes R.P., Horan T.C., Edwards J.R., Jarvis W.R., Tolson J.S., Henderson T.S., Martone W.J. (1991). Nosocomial infections in elderly patients in the United States, 1986–1990. Am. J. Med..

[88] Gould I. (2006). Costs of hospital-acquired methicillin-resistant Staphylococcus aureus (MRSA) and its control. Int. J. Antimicrob. Agents.

[89] Zhang X.-y., Huang X.-B., Jiang L., Ma Y., Fan A.-L., Tang B. (2012). Antibacterial property of Cu modified stainless steel by plasma surface alloying. J. Iron Steel Res. Int..

[90] Allion-Maurer A., Saulou-Berion C., Briandet R., Zanna S., Lebleu N., Marcus P., Raynaud P., Despax B., Mercier-Bonin M. (2015). Plasma-deposited nanocomposite polymer-silver coating against Escherichia coli and Staphylococcus aureus: Antibacterial properties and ageing. Surf. Coat. Technol..

[91] Jiang H., Manolache S., Wong A.C.L., Denes F.S. (2004). Plasma-enhanced deposition of silver nanoparticles onto polymer and metal surfaces for the generation of antimicrobial characteristics. J. Appl. Polym. Sci..

[92] Dong Y., Li X., Tian L., Bell T., Sammons R., Dong H. (2011). Towards long-lasting antibacterial stainless steel surfaces by combining double glow plasma silvering with active screen plasma nitriding. Acta Biomater..

[93] Baba K., Hatada R., Flege S., Ensinger W., Shibata Y., Nakashima J., Sawase T., Morimura T. (2013). Preparation and antibacterial properties of Ag-containing diamond-like carbon films prepared by a combination of magnetron sputtering and plasma source ion implantation. Vacuum.

[94] Sarghini S., Paulussen S., Terryn H. (2011). Atmospheric pressure plasma technology: A straightforward deposition of antibacterial coatings. Plasma Process Polym..

[95] Ibis F., Oflaz H., Ercan U.K. (2016). Biofilm Inactivation and prevention on common implant material surfaces by nonthermal DBD plasma treatment. Plasma Med..

[96] Rocha J.d.L., Pereira R.d.S., de Oliveira M.C.L., Antunes R.A. (2019). Investigation on the relationship between the surface chemistry and the corrosion resistance of Electrochemically Nitrided AISI 304 stainless steel. Int. J. Corros..

[97] Cadosch D., Chan E., Gautschi O.P., Simmen H.P., Filgueira L. (2009). Bio-corrosion of stainless steel by osteoclasts—in vitro evidence. J. Orthop. Res..

[98] Guastaldi F.P., Yoo D., Marin C., Jimbo R., Tovar N., Zanetta-Barbosa D., Coelho P.G. (2013). Plasma treatment maintains surface energy of the implant surface and enhances osseointegration. Int. J. Biomater..

[99] Benčina M., Iglič A., Mozetič M., Junkar I. (2020). Crystallized TiO_2_ Nanosurfaces in Biomedical Applications. Nanomaterials.

[100] Hsu C.-W., Chen T.-E., Lo K.-Y., Lee Y.-L. (2019). Inhibitive Properties of Benzyldimethyldodecylammonium Chloride on Microbial Corrosion of 304 Stainless Steel in a Desulfovibrio desulfuricans-Inoculated Medium. Materials.

[101] Arciola C.R., Caramazza R., Pizzoferrato A. (1994). In vitro adhesion of Staphylococcus epidermidis on heparin-surface-modified intraocular lenses. J. Cataract Refract. Surg..

[102] Taglietti A., Dacarro G., Barbieri D., Cucca L., Grisoli P., Patrini M., Arciola C.R., Pallavicini P. (2019). High bactericidal self-assembled nano-monolayer of silver sulfadiazine on hydroxylated material Surfaces. Materials.

[103] Sabino R.M., Kauk K., Madruga L.Y., Kipper M.J., Martins A.F., Popat K.C. (2020). Enhanced hemocompatibility and antibacterial activity on titania nanotubes with tanfloc/heparin polyelectrolyte multilayers. J. Biomed. Mater. Res. Part A.

